# Strategic information is everyone’s business: perspectives from an international stakeholder meeting to enhance strategic information data along the HIV Cascade for people who inject drugs

**DOI:** 10.1186/s12954-015-0073-y

**Published:** 2015-10-16

**Authors:** Richard D. Pierce, Jennifer Hegle, Keith Sabin, Edo Agustian, Lisa G. Johnston, Stephen Mills, Catherine S. Todd

**Affiliations:** 85 Sukhumvit Soi 13, #5B, Bangkok, Thailand; FHI 360, 359 Blackwell Street, Suite 200, Durham, NC 27701 USA; Joint United Nations Programme on HIV/AIDS (UNAIDS), 20, Avenue Appia, Geneva, 27 Switzerland; Persaudaraan Korban Napza Indonesia (PKNI)/Indonesian Drug User Network, Jalan Tebet Timur Dalam XI No. 94 Tebet, Jakarta Selatan, 12820 Indonesia; 64 Nieuwezijds Voorburgwal, Amsterdam, SC1012 Netherlands; FHI 360 Asia-Pacific Regional Office, 19th floor, Sindhorn Building, 130-132 Wittayu Road, Bangkok, 10330 Thailand

**Keywords:** People who inject drugs, HIV, Harm reduction, Strategic information, Key populations, Intervention, HIV Cascade

## Abstract

People who inject drugs (PWID) are at increased HIV transmission risk because of unsafe injecting practices and a host of other individual, network, and structural factors. Thus, PWID have a great need for services within the Cascade of HIV prevention, diagnosis, care, and treatment (HIV Cascade). Yet the systems that monitor their progress through the Cascade are often lacking. Subsequently, fewer reliable data are available to guide programs targeting this key population (KP). Programmatic data, which are helpful in tracking PWID through the Cascade, also are limited because not all countries have harm reduction programming from which to estimate Cascade indicators. Also, due to stigma and the illegal nature of drug use, PWID may not disclose their drug use behavior or HIV status when accessing services. Consequently, PWID appear to have low HIV testing rates and, for those living with HIV, lower access to health services and lower viral suppression rates than do other KP groups. This commentary, based on outcomes from an international stakeholder meeting, identifies data gaps and proposes solutions to strengthen strategic information (SI), the systematic collection, analysis, and dissemination of information, to optimize HIV prevention, care, and treatment programming for PWID.

## Commentary

### Introduction

Injecting drug use is the most efficient mode of transmitting HIV and viral hepatitis, and people who inject drugs (PWID) are disproportionately affected by HIV because of behaviors influenced by individual, network, and structural factors [1–3]. However, stigma and criminalization of drug use and the attendant difficulty measuring these factors confound efforts to compile much-needed HIV epidemiologic data among PWID, including prevalence, testing frequency, and, for PWID diagnosed with HIV, transition into care and treatment retention [4–6]. This lack of strategic information (SI)—defined as the systematic collection, analysis, and dissemination of information to optimize programming—hinders our ability to design effective programming interventions for, and address the needs of, PWID. Deficiencies in SI and attendant weaker programming will likely impede progress toward the UNAIDS “90-90-90” targets by 2020, which are that 90 % of all people living with HIV (PLHIV) will know their HIV status, 90 % of all PLHIV will receive sustained antiretroviral therapy (ART), and 90 % of those receiving ART will have achieved viral suppression [7].

To identify gaps in this process and recommend approaches to optimize SI collection and use for PWID programming, the meeting “People Who Inject Drugs: Strategic Information to Reach the 90-90-90: A Global Conversation to Review the Evidence And Recommend Improved SI Practices to Inform The Cascade” was convened. This commentary conveys key meeting points and recommendations from a variety of perspectives, with the goal of rectifying SI deficiencies, identifying innovative data-driven programming approaches, and integrating respectful practices into PWID SI and programmatic activities to optimize achievement of 90-90-90 goals for PWID.

### Setting

Fifty-one subject matter experts in PWID advocacy, harm reduction, and HIV surveillance and monitoring met in Bangkok, Thailand, on May 15, 2015. The meeting was hosted by LINKAGES, a global project funded by the United States Agency for International Development (USAID) and the President’s Emergency Plan For AIDS Relief (PEPFAR) and dedicated to key populations (KPs)—PWID, sex workers, men who have sex with men, and transgender people [8]. This meeting was a satellite meeting to the UNAIDS and WHO-convened Third Global HIV Surveillance Consultation (May 12–14) and all attendees were invited, as were harm reduction implementers and PWID advocacy group representatives identified by meeting organizers and LINKAGES technical staff. Attendees represented a wide variety of groups, including donor organizations (e.g., Global Fund to Fight AIDS, Tuberculosis and Malaria, USAID), United Nations agencies (UNAIDS, UNODC), non-government organizations, harm reduction-implementing organizations, academia, and national, regional, and global PWID advocacy groups; most of the latter two groups were from Asia.

The interactive meeting comprised a review of gaps in epidemiologic data among PWID and a formal presentation on SI limitations and population size estimate approaches, followed by panel discussions addressing (1) PWID community involvement in SI collection and use and (2) challenges/solutions to indicator choices and use of programmatic data to inform indicators from an implementers’ perspective. Meeting participants then divided into four groups to discuss gaps and potential solutions to SI content relevant to PWID (Table 1). The proceedings were transcribed, and main points are summarized in this article; no other documents were produced.

**Table 1:** Key strategic information gaps and proposed solutions for people who inject drugs identified within small group discussion

Group number	Discussion topic	Identified gaps	Recommendations
1	Identifying, reaching, and testing people who inject drugs (PWID)	● Populations of females who inject drugs are underrepresented	● Programs should also be able to identify emerging risk behaviors; they could gather information in both formal and informal ways
		● Population size estimates are not accurate	● Separate Cascades would allow data collectors to capture subgroups within the larger PWID groups
		● Delays in estimating population size within surveys are slowing testing and uptake because services are not provided where PWID are located	● Use programs more strategically for data collection along the Cascade
		● Reach of programs depends on political and legal environments. There is limited information from closed settings, such as prisons	● Use peer educators and PWID drop-in centers for care and support
			● Link with hospitals and other treatment sites for referrals
2	Testing PWID and enrolling them in care and treatment	● Limited data on how many PWID living with HIV are actually in care and treatment	● Community-based testing should be scaled up
		● Lack of linkages between PWID-focused programming and HIV care and treatment programming	● Scale up the use of HIV rapid tests using oral fluid samples, which are more convenient and preferable to most people
		● Stigma and discrimination prevent members of key populations from seeking testing services	● Periodically test MMT clients for HIV when they receive their methadone
		● Challenges in ensuring anonymity when tracking people along the Cascade	● Use a mix of different approaches, different entry points into the Cascade in SI collection
		● The testing service delivery model could be more targeted	● Mix program and surveillance, community support, and case management
3	Gaps in strategic information; challenges and solutions for retention of PWIDs in care and treatment	● Uneven coverage of drug dependence treatment and OST	● Integrated MMT and ART services
		● Uneven coverage of peer/family/social support	● Establish models of peer-administered ART delivery or limited peer-peer interventions to improve adherence
		● Difficult to disaggregate data by risk behavior or key population group	● Ask clinicians to collect risk group/behavior status
		● Information by risk group is not used to inform the Cascade	● In IBBS/surveys, include biomarkers for ART use in testing and data collection to inform ART uptake and viral suppression pillars within the Cascade
		● Lack of data on reasons for loss to follow-up and on mortality	
4	Respectful approaches to data collection	● “Respectful” can mean different things to different people	● Protect PWID identities through encryption and unique identifier codes
		● There are different clearance requirements for different countries	● Adopt the human rights framework for SI activities
		● The meaning of community can vary, and this can challenge how PWID communities are integrated in data collection	● Consider the community-based participatory research framework
		● It can be difficult to guarantee anonymity	● Ensure that services are available for the kinds of problems that are being investigated. For example, needle distribution and MMT programming should be identified before initiating a study that intends to refer participants to these services upon request
		● How to get people to disclose behaviors/practices that are criminalized	● Require funders of research to accept responsibility for guaranteeing anonymity

### Controversies and gaps in HIV epidemiology among PWID

Current global HIV prevalence data among PWID belie substantial variance in sub-epidemics and in national reporting approaches. Many countries do not submit single national HIV prevalence figures for some or all KPs, and some countries do not have updated data for international analysis [9]. National prevalence data are typically gathered from integrated bio-behavioral surveys (IBBS), which are conducted in areas, usually large cities, with known high rates of drug use. Other methods of obtaining prevalence data include sentinel surveillance and case reporting. As a result, these estimates may not reflect the entire country or account for emerging epidemics. For example, the Kenyan IBBS in Nairobi and Mombasa, cities with documented large drug user populations, reported HIV prevalence of 30 % among PWID. However, in Kisumu, Kenya, which was not included in national data collection, ethnographic research detected a new, hidden population of PWID among whom HIV prevalence was 19 % [10]. Therefore, it may not be wholly accurate to rely solely on national-level data for HIV prevalence among PWID or to make programming and funding decisions without considering local and regional trends in HIV prevalence and risk behaviors.

Similar issues are present for estimating PWID population sizes, as regional and global estimates are impacted by outdated or insufficient data. Forty countries included in a 2014 global harm reduction assessment did not provide national PWID population size estimates within the last 8 years. By region, Eurasia was found to have some of the strongest reporting and service delivery programs, while, in Asia, 60 % of 25 reporting countries submitted outdated figures, in some cases from 2001 [11]. This lack of recent data raises two concerns: that neither meaningful HIV data collection on PWID nor HIV prevention among PWID are political priorities within these countries.

### Choosing the best SI approach

Data collection methods to inform HIV programming for PWID must be scientifically robust and context- and population-appropriate. For example, locations with small PWID populations, but potentially explosive epidemics, are not well represented within IBBS. Further, specific sub-populations of PWID, such as adolescent and young PWID and females who inject drugs are poorly represented within IBBS, even when using chain-referral sampling methods. At the meeting, presenters suggested conducting targeted surveys for these vulnerable groups as well as including youth in future adult studies by broadening age eligibility. Though parental consent is necessary in many settings, many countries are reducing the age requiring parental consent for HIV testing and counseling, which could open an effective avenue for countries to allow surveys among adolescent KPs.

Female PWID also are often under-represented in some SI approaches, such as surveys using respondent-driven sampling (RDS). Women who inject drugs tend not to have robust social networks, compared to men, and are often excluded from analysis. As a result, little or no data are collected about the magnitude of their HIV burden. In Vietnam, females were excluded in both sentinel surveys and the 2012 IBBS due to sampling barriers, and in Myanmar, RDS referral chains often terminated at female participants. New strategies, or perhaps more qualitative data, are thus needed to collect actionable SI from women who inject drugs.

Case reporting or collection of harm reduction programmatic data may be more feasible as it allows for the collection of local level data that are eventually reported to national-level health officials [12]. Methodologic questions to consider when selecting an SI approach are: “What is the definition of PWID and how does this change by context?” “Will it be regional or ‘hot-spot-based’ within countries?” and “What are the data sources and are data quality and focus appropriate?” However, responses to these diverse HIV epidemics require denominators to measure the number of people living with HIV for a given KP, a pillar in the HIV Cascade. One way to estimate these denominators, as well as inform other Cascade components, is to ask participants about their HIV status in IBBS or similar surveys [13]. Consistent concerns collecting SI data include sensitivity in asking people about their drug use and HIV status and assuring confidentiality in reporting them, especially given the potential to further criminalize and stigmatize this population.

To reach 90-90-90 goals, SI systems for PWID need to move to a “treatment-oriented” approach, increasing the value of case-based reporting and following cases through the HIV Cascade. Case-based reporting raises confidentiality concerns as reporting requires following individuals “through the system” and necessitates the use of unique identifiers. Another option for obtaining data for monitoring the HIV Cascade is to use programmatic harm reduction data. However, program data does not perfectly overlap with HIV Cascade indicators; for example, HIV testing may occur within harm reduction programs, but care and treatment services are provided at separate sites with no formal data linkage to allow follow-up through the Cascade. Double counting is also an issue, which could be overcome by using unique identifiers and recall of last testing site and date, with attendant confidentiality concerns.

### Collaborative approaches to integrating PWID in SI data selection and collection

Within the “90-90-90” initiative, respect for human rights is prominent—including involvement of KP groups in collecting SI. PWID are the best resource for learning how to provide services to peers, and their involvement in data collection is critical for ensuring a rights-based approach. Based on their personal experience with criminalization and stigma associated with drug use and further HIV-associated stigma, PWID advocacy group representatives at the meeting recommended that they be treated as partners in, rather than just subjects of, research and SI data collection.

One way to ensure collaboration is to improve the capacity of community-based, constituency-led organizations to: engage in survey data collection, analysis, and dissemination; documentation; and monitoring and evaluation. A successful example of this principle in practice is the *Persaudaraan Korban Napza Indonesia* (PKNI)/Indonesian Drug User Network. Beginning in 2007, PKNI, in collaboration with Oxford University, initiated *Perempuan Bersuara*/“Women Speak Out,” a cross-sectional study among approximately 700 females who inject drugs to investigate factors associated with HIV risk among this subgroup. PKNI established a community advisory group comprising females who inject drugs to inform questionnaire design and guide ethical and operational study aspects. The community-led team received ongoing capacity building in research methods and data interpretation from researchers, allowing them to develop marketable skills and knowledge while playing a significant role in the study’s implementation. This collaborative approach resulted in a strong sense of community ownership of the research and active participation from female respondents in the project.

Criminalization of drug use is a major barrier to collaboration with PWID. In Indonesia, for example, the inclusion of PWID in SI collection is affected by a new “War on Drugs.” Between 2014 and 2015 in Jakarta, Bogor, and Bandung, community interviewers reported harassment by National Narcotics Board officers and the police, including raids, random urine testing, and arrest threats during interviews. Such incidents led to lower recruitment rates because of increased distrust and fear by participants of providing information to researchers, potentially resulting in reporting bias and less reliable outcomes.

### Novel models for improved SI collection and utilization

Capturing data to inform all Cascade indicators is challenging and involves aggregation of clinical data up to the national level. This requires a mechanism for centralization of data and consistency between data collection methods across multiple programs. Two supply-side intervention models to improve HIV testing uptake, diagnosis, and enrollment in care, specifically through increasing numbers of PWID reached and tested, were presented from Vietnam. In urban areas, the “Fansipan Challenge” model recruited opiate substitution therapy clients, most of whom inject, to bring friends or sexual partners for HIV testing, and those testing positive to be enrolled in care and treatment. This model rewards clients for each referral completing testing and subsequent referrals with points and phone credit, structured as a contest to climb Fansipan, Vietnam’s highest mountain [14]. For rural areas, a direct referral model was tested in mountainous provinces near the Lao border, where drug trafficking and injecting use are common and care and treatment commensurately low. This model offers hamlet health worker incentives for successful referral for mobile and facility-based HIV counseling and testing, identification of HIV-positive individuals, and re-engaging known HIV patients who were lost to care. In both models, unique identifiers were used to track individuals across the HIV Cascade, and questionnaires included more questions (picture-based) on high-risk behaviors to provide meaningful SI contributions.

Scaling up successful program models and the accompanying SI concerns were also discussed. One panelist described the transition of harm reduction programming from small programs operated by civil society organizations to implementation of the same model by state and national government. While this transition is in process, SI and operations research has identified gaps in quality of care, through lack of or insufficient number of syringes received from needle and syringe distribution and collection programs and low ART uptake among clients living with HIV. This example of using SI to improve service quality is directed toward creating public-private partnerships and improving political will to provide quality services and enhance 90-90-90 outcomes.

### Small group discussions

This meeting also included four small group discussions on gaps related to and recommendations for SI collection for four content areas. Table 1 summarizes key gaps and recommendations. Topics assigned to each group did not allow for frequent overlap in recommendations, but several key themes were present from all groups. These themes included the following: (1) the need for integration of Cascade indicators into PWID programming, potentially with merged harm reduction and HIV management services; (2) expanding the role of peers within the PWID community to improve testing coverage; (3) linkages to and retention in care; and (4) respectful SI collection and interpretation.

### Conclusions

Reaching PWID populations for HIV prevention, diagnosis, and treatment and tracking them through the HIV Cascade of services is challenging due to persistent barriers from governments, society, and health systems. PWID are highly stigmatized, criminalized, and hidden, posing significant challenges to estimating their population sizes and HIV prevalence and assessing their prevention, care, and treatment needs. This meeting delineated these challenges and sought consensus on ways to derive more accurate data, identify data gaps related to service uptake along the HIV Cascade, and outline SI methods to better monitor and improve the prevention and treatment needs of PWID to support 90-90-90 goals. One key output was consensus around the need to use programmatic data, with the caveat that PWID-centered programs must be held accountable for reporting accuracy, potentially through assessments by a third party. There was clear consensus on the need to involve the PWID community in SI data collection.

Finally, participants agreed that harm reduction program client confidentiality must be maintained and respected. Linking data between harm reduction programs and HIV care and treatment services is critical, and models utilizing unique codes or other approaches that respect client anonymity need to be piloted and expanded if successful. The meeting concluded with final comments that SI must have direct application to program utilization and improvement, as the data are meant to serve the clients and community. The closing message from the meeting was that “SI is everyone’s business,” reflected by the importance of identifying the best surveillance methods and HIV prevention, care, and treatment program monitoring for PWID for filling the gaps in the HIV Cascade and realizing the 90-90-90 goal.
